# Recombination Drives Vertebrate Genome Contraction

**DOI:** 10.1371/journal.pgen.1002680

**Published:** 2012-05-03

**Authors:** Kiwoong Nam, Hans Ellegren

**Affiliations:** Department of Evolutionary Biology, Evolutionary Biology Centre, Uppsala University, Uppsala, Sweden; Stanford University, United States of America

## Abstract

Selective and/or neutral processes may govern variation in DNA content and, ultimately, genome size. The observation in several organisms of a negative correlation between recombination rate and intron size could be compatible with a neutral model in which recombination is mutagenic for length changes. We used whole-genome data on small insertions and deletions within transposable elements from chicken and zebra finch to demonstrate clear links between recombination rate and a number of attributes of reduced DNA content. Recombination rate was negatively correlated with the length of introns, transposable elements, and intergenic spacer and with the rate of short insertions. Importantly, it was positively correlated with gene density, the rate of short deletions, the deletion bias, and the net change in sequence length. All these observations point at a pattern of more condensed genome structure in regions of high recombination. Based on the observed rates of small insertions and deletions and assuming that these rates are representative for the whole genome, we estimate that the genome of the most recent common ancestor of birds and lizards has lost nearly 20% of its DNA content up until the present. Expansion of transposable elements can counteract the effect of deletions in an equilibrium mutation model; however, since the activity of transposable elements has been low in the avian lineage, the deletion bias is likely to have had a significant effect on genome size evolution in dinosaurs and birds, contributing to the maintenance of a small genome. We also demonstrate that most of the observed correlations between recombination rate and genome contraction parameters are seen in the human genome, including for segregating indel polymorphisms. Our data are compatible with a neutral model in which recombination drives vertebrate genome size evolution and gives no direct support for a role of natural selection in this process.

## Author Summary

One major implication from genetic work done several decades ago is that the genome contains a lot of sequences that do not constitute genes or other functional elements. The total amount of DNA—the genome size—is thus not necessarily an indicator of DNA complexity or organismal complexity, an observation often referred to as the C-value paradox (C-value being a measure of DNA content). What then is it that determines genome size? One model posits that the evolution of genome size is not a consequence of natural selection but is instead governed by the incidence and character of naturally occurring mutations that affect the length of DNA, a process that is not affected by selection. Here we present the results of an analysis of how recombination affects the size of avian and human genomes. We find strong evidence that the rate of recombination is a driving force of genome size evolution. In regions of the genome where recombination occurs frequently, the loss of DNA caused by small deletions is particularly pronounced. Our simulations show that the effect of such recombination-driven genome contraction can be profound over evolutionary time scales. These observations lead to a model in which recombination is mutagenic for length changes and that the incidence of deletions increases with increasing recombination rate. Although we cannot formally exclude that natural selection contributes to the observed relationship between recombination and genome contraction, we find no evidence to support such a scenario.

## Introduction

A link between the dynamics of intron evolution and recombination has been found in form of a negative relationship between recombination rate and intron size, seen in Drosophila [1], [2], humans [2] and chicken [3]. Two hypotheses based on natural selection have been proposed to explain this relationship. First, insertion mutations increasing intron length, which may confer higher energy cost for transcription or replication and thus be mildly deleterious, may be more efficiently removed by purifying selection in regions with high recombination rate where Hill-Robertson interference is reduced [1]. Second, insertion mutations increasing intron length may be favored in regions with low recombination rate because large introns reduce the effect of Hill-Robertson interference [4].

The negative relationship between intron length and recombination could also be possible to explain under a neutral scenario if recombination itself, either directly (by being mutagenic) or indirectly (by affecting other genomic features) affects the direction or magnitude of changes in intron length. More generally, a mutational bias associated with recombination that leads to increases or decreases in sequence length all over the genome (and not only in introns) will have implications to the overall DNA content, i.e., the evolution of genome size. Several models for genome size evolution have been presented. Broadly speaking they can be defined as adaptive [5]–[8], non-adaptive [9] or neutral [10], [11]. Short deletions are almost ubiquitously found to outnumber short insertions in eukaryotic genomes and it has been proposed that the degree of deletion bias is a main factor for variation of genome size under a neutral model [10], [11]. Recombination-associated processes can potentially provide a mechanistic explanation to the deletion bias, which remains to be tested. Taking possible mutagenic effect of recombination into account is clearly necessary before inference on selection from correlation between recombination rate and sequence length is made.

In this study we address the underlying evolutionary forces that contribute to a negative relationship between recombination rate and sequence length by focusing on three sequenced and annotated vertebrate genomes from two major lineages, mammals (human) and birds (chicken and zebra finch). Detailed recombination rate maps are available for all these species [12]–[14]. Avian genomes are typically smaller than mammalian genomes; 75% of >400 characterized bird species have a haploid DNA content of 1.2–1.6 pg, whereas 75% of >600 characterized mammalian species have 2.5–4.3 pg [15]. A focus on avian genomes is of particular interest in the context of genome size evolution in relation to recombination because both chicken and zebra finch display an unusual heterogeneity in the rate of recombination, including recombination-prone microchromosomes [3] and a stronger “telomere-effect” (elevated recombination rates toward chromosome ends) than so far seen in any other species [13], [14]. Potentially, such heterogeneity can increase the power in detecting correlations between recombination rate and other genomic parameters. In addition, because avian genomes show a high degree of karyotype and synteny conservation [16], [17], genomic correlates may be less affected by noise following from frequent chromosomal rearrangements.

Using comparative genomics to analyze structural variation in non-coding DNA is usually limited by the problem of aligning sequences evolving under low or no constraint in other than closely related species. Moreover, unless sequence data can be aligned from three or more species, it is impossible to distinguish insertions from deletions. Furthermore, if insertions or deletions occur in genomic regions containing functional elements [18], selection may act differently on the two types of structural changes. Here we circumvent these problems by using transposable elements contained within non-coding DNA to measure insertion and deletion rates in individual lineages. Specifically, we infer insertion and deletion events from alignments of repeat elements with their ancestral master sequence, as introduced by Petrov et al. [19]. Our main observation, consistent across all three species, is that loss of DNA is most pronounced in regions of high recombination. This is compatible with a neutral model of genome evolution where recombination drives genome contraction.

## Results

### Transposable elements, sequence length, and recombination rate in avian genomes

We identified Long Interspersed Elements (LINEs) from pre-masked genome assemblies of chicken and zebra finch using Repeatmasker. A total of 239,812 (chicken) and 169,576 (zebra finch) LINEs were found, the far most abundant type being the well-known CR1 retroposon [20]–[23]. Using data from 1 Mb non-overlapping windows across the genome, we found a significant negative correlation between recombination rate and intron length in both species (chicken, τ = −0.18, *p*<0.001; zebra finch τ = −0.14, *p*<0.001; Kendall's rank test) (Table 1), and this was also the case when only first introns were considered in chicken (τ = −0.12, *p*<0.001) but not in zebra finch (τ = −0.04, *p* = 0.120). Moreover, there was a significant negative relationship between recombination rate and the length of individual LINE sequences located within introns (chicken, τ = −0.32, *p*<0.001; zebra finch, τ = −0.40, *p*<0.001) as well as between recombination rate and the length of intronic sequence that is not LINE sequence (chicken, τ = −0.16, *p*<0.001; zebra finch, τ = −0.13, *p*<0.001) (Table 1). This shows that if the rate of LINE integration is higher in regions with low recombination rate, it cannot fully explain the negative relationship between intron length and recombination rate. Furthermore, there was a significant negative correlation between recombination rate and the length of intergenic sequence (intergenic spacer) in both species (chicken, τ = −0.32, *p*<0.001; zebra finch, τ = −0.15, *p*<0.001) and a positive correlation between recombination rate and gene density (chicken, τ = 0.30, *p*<0.001; zebra finch τ = 0.16, *p*<0.001) (Table 1). All these observations point at a pattern of more condensed structure in regions of high recombination in avian genomes.

**Table 1:** Strength (correlation coefficient, τ) and statistical significance (*p*) of Kendall's rank correlations between recombination rate and various genomic parameters in non-overlapping 1 Mb windows.

	Chicken		Zebra finch		Human	
	τ	*p*	τ	*p*	τ	*p*
Intron length	−0.18	<0.001	−0.14	<0.001	0.03	0.061
First intron length	−0.12	<0.001	−0.04	0.120	0.05	0.001
Length of individual LINEs	−0.32	<0.001	−0.40	<0.001	−0.16	<0.001
Length of unique sequence within introns	−0.16	<0.001	−0.13	<0.001	0.05	<0.001
Intergenic spacer length	−0.32	<0.001	−0.15	<0.001	−0.03	0.061
Length of unique sequence within intergenic regions	−0.26	<0.001	−0.11	0.007	0.04	0.052
Gene density	0.30	<0.001	0.16	<0.001	0.02	0.159

### Rates of insertion and deletion and their relationship with recombination rate

**Figure 1:**
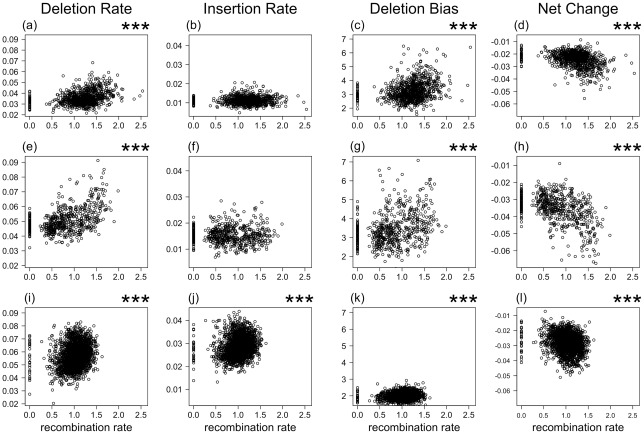
The relationship between recombination rate (x-axis, fourth-root) and deletion rate, insertion rate, deletion bias (deletion rate/insertion rate), and rate of net sequence length change (insertion rate – deletion rate). In chicken (a, b, c, and d), zebra finch (e, f, g, and h), and human (i, j, k, and l). *p*<0.001 in Kendall's rank correlation test is depicted by ***.

We estimated insertion and deletion rates by aligning repeat elements with their master sequence and by inferring events of small insertion and deletion from gaps in master and repeat element sequence, respectively (see Figure S1 for distribution of the length of insertions and deletions). The rate of deletion defined as the number of bp deleted per bp repeat (LINE) sequence was consistently higher than the rate of insertion, giving a deletion bias of 3.24 and 3.45 in chicken and zebra finch, respectively (Table 2). Using data from 1 Mb windows, there was a significant positive correlation between recombination rate and deletion rate (chicken, τ = 0.23, *p*<0.001; zebra finch, τ = 0.32, *p*<0.001) (Figure 1), but no correlation between recombination rate and insertion rate (chicken, τ = −0.01, *p* = 0.776; zebra finch, τ = −0.03, *p* = 0.323). There was also a positive correlation between recombination rate and the number of deletion events (chicken, τ = 0.37, *p*<0.001; zebra finch, τ = 0.38, *p*<0.001).

**Table 2:** The mean rate of substitution, insertion, and deletion (as the number of bp inserted or deleted per bp repeat sequence) for LINEs in the genomes of chicken, zebra finch, and human.

Species	Substitution rate	Insertion rate	Deletion rate	Deletion bias
Chicken	0.255 (0.252–0.257)	0.0112 (0.0111–0.0113)	0.0356 (0.0351–0.0359)	3.24 (3.19–3.29)
Zebra finch	0.357 (0.354–0.360)	0.0154 (0.0151–0.0156)	0.0509 (0.0502–0.0516)	3.45 (3.38–3.52)
Human	0. 343 (0.341–0.346)	0.0285 (0.0283–0.0286)	0. 0565 (0.0560–0.0568)	1.99 (1.98–2.00)

Rate (divergence) estimates are based on sequence alignments of individual repeat elements and their master sequence.

Insertions and deletions taken together, and not surprisingly given the above correlations, there was a positive relationship between recombination rate and the deletion bias (chicken, τ = 0.19, *p*<0.001; zebra finch, τ = 0.24, *p*<0.001) (Figure 1). While this suggests genomic contraction in high recombination regions, such trend could in theory be mitigated by relatively short insertion and deletion events (although the deletion bias being high) in high recombination regions. However, recombination rate was as strongly correlated with the net change in sequence length (amount of sequence deleted minus amount of sequence inserted; chicken, τ = 0.24, *p*<0.001; zebra finch, τ = 0.33, *p*<0.001) (Figure 1) as it was to the deletion bias calculated on basis of rates of insertion and deletion. We thus conclude that there has been a process of genomic contraction in high recombination rate regions during avian evolution. This process is manifested in a more condensed present-day genomic structure in regions with high recombination.

In the above we have assumed a star phylogeny for the relationship among repeat copies and their master sequence. To exclude the possibility that violation of this assumption would affect our conclusions we repeated the analyses only using insertions and deletions that were seen once. This replicated the correlations seen with the whole data set (deletion bias: chicken, τ = 0.09, *p*<0.001; zebra finch, τ = 0.08, *p*<0.001; net change in sequence length: chicken, τ = −0.23, *p*<0.001; zebra finch, τ = −0.22, *p*<0.001).

All relationships reported above are for 1 Mb genomic regions, which is the smallest window size for which we have resolution in data on the regional recombination rate. Since recombination in at least mammalian genomes is often concentrated to narrow hot spot regions [24], more fine-scale recombination maps could potentially have given stronger correlations between recombination and parameters of genome contraction. On the other hand, primate recombination hot spots tend to be ephemeral with a rapid turnover rate [25], [26] and this may obscure correlations with genomic parameters representing mutational events that have accumulated over long evolutionary time scales. While we are not able to analyze smaller windows we repeated all analyses using 5 Mb windows. Interestingly, most parameters related to genome contraction showed stronger correlation with recombination rate for this window size than for 1 Mb windows (Table S1). For example, Kendall's τ was as high as −0.43 and −0.53 for the correlation with the length of individual LINEs and 0.34 and 0.47 for the correlation with the net change in sequence length in chicken and zebra finch, respectively.

### Comparison between sex chromosome and autosomes

As a specific test of the role of recombination in affecting genome size evolution we compared sequences on autosomes and sex chromosomes. Birds have female heterogamety (males ZZ, females ZW) so the Z chromosome does only recombine in males and thus have a lower recombination rate than autosomes. If recombination drives genome contraction we expect the deletion bias to be higher on autosomes than on the Z chromosome and the mean length of LINEs to be longer on the Z chromosomes than on autosomes. Data from both chicken and zebra finch meet these expectations. The deletion bias was significantly lower on the Z chromosome than on the autosomes (chicken, 3.23 vs. 3.32, *p* = 0.045; zebra finch, 3.19 vs. 3.74, *p*<0.001, 10,000 times of non-parametric bootstrapping based on stratified sampling) and the mean length of LINEs was longer on the Z chromosome than on autosomes (chicken, 419.8 bp vs. 314.5 bp; zebra finch 384.6 bp vs. 266.6 bp, *p*<0.001 in both species).

The pattern for the non-recombining, female-specific W chromosome should be expected to differ even more from that of autosomes. The W chromosome is not included in the zebra finch assembly and the amount of W-linked sequence in the chicken assembly is limited (0.26 Mb). However, the chicken W chromosome had the longest mean length of LINEs (446.7 bp, significantly different from autosomes, *p*<0.001) and the least pronounced deletion bias (2.38, *p*<0.001).

### Chromosome size per se does not explain the relationship between recombination rate and genome contraction parameters

Recombination rate is closely correlated with chromosome size in both chicken [3] and zebra finch [27]. In theory, it is possible that some other genomic parameter that also correlates with chromosome size causes the observed relationships between recombination rate and different attributes of genome contraction (e.g., intron length, deletion rate and deletion bias). To test this possibility we used a mixed model with chromosome identity as a random variable. However, the majority of the observed correlations between recombination rate and parameters associated with genome contraction remained statistically significant when chromosome identity was controlled for (Table S2).

Another way of excluding possible effects of chromosome identity is to study the relationship between recombination rate and deletion rate/deletion bias for individual chromosomes (Table S3). For the microchromosomes the number of available windows is not sufficient for this analysis and we thus restricted the analysis to chromosomes with at least 20 windows (i.e. chromosomes >20 Mb in size). Nine out of 11 such chromosomes in chicken showed a positive correlation (mean Kendall's τ = 0.11) between recombination rate and deletion rate (randomization test with 10^6^ replicates, *p* = 0.033) and 10 out of 11 chromosomes showed a positive correlation (mean τ = 0.11) between recombination rate and the deletion bias (*p* = 0.006). In zebra finch, eight out of eight chromosomes had a positive correlation (mean τ = 0.22) between recombination rate and deletion rate (*p* = 0.004), and seven out of eight had a positive correlation (mean τ = 0.12) between recombination rate and the deletion bias (*p* = 0.035). The genome-wide relationships between recombination rate and genome contraction parameters can thus also be seen within individual chromosomes.

### The impact on avian genome size variation

We simulated the impact of deletion-biased length mutations on avian genome size evolution over time by fitting an exponential decay function based on the assumptions of a constant rate of sequence loss and neutral evolution. We used sequence divergence (rather than years) as a time scale to avoid uncertainties associated with rate calibration of the molecular clock; this is particularly warranted given apparent heterotachy in avian substitution rates [28]. From 8,328 ancestral CR1 sequences identified in whole-genome alignment of chicken, turkey, and zebra finch [29], we estimated rates of sequence evolution in the chicken branch as follows: substitution rate 4.21% (95% confidence interval, CI: 4.01–4.45%), deletion rate 2.61% (1.99–3.33%), and insertion rate 0.58% (0.52–0.65%). Combining these three estimates, this translates into a loss of 0.48 (0.34–0.60) nucleotides per nucleotide substitution. The rate parameter of this exponential decay was 0.489 (0.347–0.664; see Methods).

To get an idea of the estimated effect of the deletion bias on avian genome size evolution we note that lineage-specific divergence (nucleotide substitutions) in the chicken lineage subsequent to the split between birds and lizards has been estimated to 0.411 [30]. If we assume a constant rate parameter of sequence loss (0.489), the chicken genome has lost sequences corresponding to 18.2% of the total DNA content (95% CI: 13.3–23.9%; Figure S2) due to small insertions and deletions since the common ancestor of birds and lizards. This assumes that the rate and pattern of indel mutations observed within transposable elements are representative for the whole genome. In the comparison of short (1–2 bp), intermediate (3–20 bp) and large (>20 bp) indel events in our data, the intermediate size category has had the largest influence on genome size change (Figure S3). Note that the estimated loss of DNA may at least in part have been balanced by gain of DNA due to large-scale insertions.

### Recombination also correlates with the deletion bias in the human genome

To test if genome compaction driven by recombination is widespread among vertebrates we analyzed data from a total of 1,724,413 LINEs in the human genome (Table 1). Similar to what was seen in birds, the deletion bias in the human genome was positively correlated with recombination rate (τ = 0.07, *p*<0.001; τ = 0.07, *p* = 0.001 using only unique events), although the bias was less pronounced (1.98). Recombination rate was positively correlated with deletion rate (τ = 0.20, *p*<0.001; Figure 1) and in this case also with insertion rate (τ = 0.16, *p*<0.001). The net change of sequence length was negatively correlated with recombination rate (τ = −0.17, *p*<0.001; τ = 0.03, *p* = 0.047 using only unique events). As for the avian data, these correlations remained statistically significant when chromosome identity was controlled for (Table S2). Moreover, when individual chromosomes were analyzed separately, 19 out of 22 chromosomes showed a positive correlation (mean Kendall's τ = 0.13) between recombination rate and deletion rate (randomization test with 10^6^ replicates, *p*<0.001) and 17 out of 22 chromosomes showed a positive correlation (mean τ = 0.06) between recombination rate and the deletion bias (*p* = 0.001) (Table S3). In summary, the patterns of insertion and deletion seen in the two avian genomes were largely replicated by data from the human genome.

### Human polymorphism data give no support that selection would explain the link between recombination and the deletion bias

All the observations made above are consistent with a neutral model in which recombination promotes deletion. Could they also be compatible with a model invoking a role of selection? Selection is more efficient in regions of high recombination and slightly deleterious alleles are therefore expected to accumulate at a lower rate (and advantageous alleles at a higher rate) in such regions. However, it may be difficult to imagine a scenario where recombination rate would correlate positively with the deletion bias due to an increased fixation probability of deletions within transposable elements, or decreased fixation probability of insertions, in high recombination regions. This would require that small indels within LINEs are not selectively neutral (or that there is differential selection for insertions and deletions; see below) but Lunter et al. [31] showed that the distribution of insertions and deletions in ancestral repeats shared between human and mouse is consistent with a neutral model and Petrov and colleagues [19], [32] have convincingly argued against purifying selection acting on indels in dead-on-arrival elements in Drosophila.

If recombination promotes deletions by being mutagenic, rather than via selection and altered fixation probabilities of indels, we should expect to see a correlation between recombination rate and the deletion bias in within-species polymorphism data. There is no large-scale data on polymorphic indels in birds but Mills et al. [33] reported nearly 2 million segregating indels in the human genome. These polymorphisms are mostly from unique sequence given the difficulty to confidently map short next-generation sequencing reads to repeat elements. Insertions were distinguished from deletions by comparison to chimpanzee outgroup sequence. We found that there was a significant positive correlation between recombination rate and the deletion bias among polymorphic human indels (τ = 0.08, *p*<0.001), and this holds true also when introns (τ = 0.06, *p*<0.001) and intergenic sequence (τ = 0.06, *p*<0.001) were analyzed separately.

As mentioned above, for a positive correlation between recombination rate and deletion bias to be seen under a selection model is required that purifying selection against insertions is more effective than purifying selection against deletions in high recombination regions. Put in other words, the deleterious effects of insertions have to be larger than those of deletions. For indels in functional regions of the genome, like protein-coding sequence, the opposite is observed in mammals and Drosophila [11], [18], [34]. We used allele frequency data from 10,003 human indels [33] to see if the site frequency spectrum differs between insertions and deletions in the genome. The spectrum is expected to be biased towards rare alleles in the presence of purifying selection, and increasingly so as the intensity of selection increases [35]. However, we found no evidence for that segregating rare alleles (minor allele frequency, MAF, <0.05) would occur more frequently among insertions than among deletions (proportion of loci with MAF<0.05 in intergenic sequence: 0.229 vs. 0.210, chi-square = 2.72, *p* = 0.099; in LINEs: 0.146 vs. 0.231, chi-square = 1.97, *p* = 0.160) (Table S4). For intronic sequence, where functional elements are more likely to be present, deletions were significantly more biased towards rare alleles than insertions (chi-square = 12.14, *p*<0.001)

## Discussion

Petrov and colleagues [10], [11] have hypothesized that the extent to which small deletions outnumber small insertions, the deletion bias, is a main factor determining genome size. This hypothesis comes mainly from the observation that in species with small genomes, the deletion bias is more pronounced than in species with larger genomes. A genomic parameter that affects the magnitude of this mutational bias could then be a driving force of the evolution of genome size under a neutral model. The same could apply to variation in compactness and chromosome size within genomes. Our data suggest that the rate of recombination represents such a parameter. Using data from two avian genomes where recombination is highly heterogeneous we find that recombination rate correlates (a) negatively with the length of introns as well as intergenic regions and with the inverse of gene density, (b) positively with the rate of deletion but negatively with the rate of insertion, and (c) positively with the deletion bias as well as the net change in sequence length. We make similar observations for the human genome, including for polymorphism data, indicating that recombination is a general factor modulating genome size variation in vertebrates. This conclusion is in line with the observation that, across species, mammalian genome size is negatively correlated with recombination rate [36].

A main criticism against the idea that the deletion bias affects genome size evolution is that the number of small deletions is too small to impact on genome size [37]. Our simulations suggest a loss of nearly 20% of the DNA content in the chicken lineage since the common ancestor of birds and lizards due to small insertions and deletions. This may very well have been sufficient to counteract genome expansion due to the spread of interspersed repeats during this period of time. Less than 10% of the chicken genome consists of recognizable transposable elements [3] and although ancient elements that have mutated beyond recognition may add to this proportion, it is clear that transposable element activity has been low in the avian lineage [3], [23]. Using bone-cell size as an indirect measure of genome size, Organ *et. al* [38] showed that the small genome size typical for contemporary birds was present already in the saurischian dinosaur lineage 230–250 million years ago [39]. We suggest that this apparent stasis of genome size through the evolution of non-avian dinosaurs and modern birds relates to a balance between moderate repeat expansion and DNA loss from the deletion bias.

Avian genomes differ from mammalian genomes in several respects, notably by being much smaller and therefore more condensly organized with shorter introns and shorter intergenic distances [40], [41]. Another avian characteristic is the significant within-genome variation in chromosome size with numerous small microchromosomes (<20 Mb). The origin and evolution of microchromosomes remains to be an enigmatic issue [42]. Although fissions and fusions are likely to be involved in generating variation in chromosome size, our results point at an interesting model for the maintenance and perhaps even further diminutivization of microchromosomes. Recombination rate correlates closely with chromosome size in avian genomes [3], a situation that follows from an obligate crossing-over per chromosome (arm) [43]. Given the observation that recombination rate correlates with the deletion bias, we propose, inspired by Burt [42], that there is a vortex where high recombination rates in small chromosomes make them even smaller due to the deletion bias, in turn leading to even higher recombination rates, etc. However, and as suggested by Petrov [11], as genome structure becomes more condensed, the likelihood for deletion events to involve functionally important sequences will increase. As a consequence, at some point selection against deleterious deletion events will occur sufficiently often to counteract quantitatively the mutational deletion bias.

Our results are compatible with that recombination by some mechanism introduce deletion mutations. While the often seen (e.g. humans, Drosophila) positive correlation between recombination rate and levels of within-species genetic diversity [44]–[47] could potentially be interpreted to reflect that recombination is mutagenic also for point mutations, recombination reduces the effect of selection at linked loci thereby acting towards maintenance of genetic variation. On the other hand, support for a neutral link between recombination and nucleotide substitution has been provided by the observation in humans and Drosophila that regions of the genome with low recombination rate also show reduced rates of between-species divergence [45], [48], [49]. However, this remains a contentious issue because several contradictory conclusions have been claimed [50]–[54].

With these uncertainties about recombination and point mutation in mind, we may ask if there is any mechanistic support for recombination being mutagenic for deletion. DNA polymerases δ and ε are key enzymes for eukaryotic DNA replication, including in connection with homologous recombination (reviewed in [55]). Both enzymes tend to cause deletions more often than insertions [56]–[59], a situation that is likely to explain the general phenomenon of deletion bias. Possibly, proofreading is less efficient to correct for unpaired bases in the primer strand than in the template strand [57]. Important in this context, DNA polymerase δ is preferentially used to promote heteroduplex extension during recombination [60]. DNA polymerase δ has lower fidelity than DNA polymerase ε, and this difference is especially pronounced for deletions. Fortune et al. analyzing *Saccharomyces cerevisiae* found that DNA polymerase δ has a 30-fold lower accuracy for large deletions and a 13-fold lower accuracy for single nucleotide deletions compared to DNA polymerase ε [57]. This may point at a mechanistic link between recombination and the rate of small deletions.

The model of recombination driving genome compactization, if correct, can explain another observation made for most investigated eukaryotic genomes: a positive correlation between GC content and gene density [61]–[67]. In both mammals and birds, GC content is one of the strongest predictors of recombination rate [28], [36]. It has been suggested that this is due to recombination driving GC-biased biased gene conversion (gBGC), a process of segregation distortion favoring the fixation of G and C nucleotides, leading to increased GC content in regions with high recombination rates [68]–[71]. If the deletion bias is more pronounced in these high recombining regions, as our data suggest, they will come to have a more compact structure with less intergenic DNA and thereby giving rise to a correlation between GC and gene density.

A general caveat in studies of the relationship between recombination and genomic parameters is that while estimates of recombination rates reflect the contemporary situation, most genomic parameters (substitution rates, base composition, chromosomal organization) are the result of long-term evolutionary processes. It follows that if regional recombination rates vary over time [72], this may obscure correlations between recombination rate and genomic parameters. However, it seems plausible that this would mostly lead to weakened correlations, not cause spurious correlations. Importantly, the recombinational landscape in birds of more conserved than in other vertebrate groups; we recently found that the recombination rate measured in 1 Mb windows are highly correlated (Spearman's rho = 0.50) between chicken and zebra finch despite these two lineages diverged 60–80 million years ago [14]. The unusually stable karyotype of birds [16], [17] is likely to contribute to this conservation.

There are at least two ways to study mutation processes using divergence data from transposable repeat elements spread across the genome. First, divergence can be estimated from alignments of ancestral (orthologous) repeats (ARs) shared by species; when AR data is available for three or more species, lineage-specific divergence can be estimated. Second, divergence can be estimated by alignments of master (consensus) and “offspring” sequences, like in the present study. Using ARs shared by human, chimpanzee and macaque, Kvikstad *et al.* [73] found that the rate of insertion, but not the rate of deletion, was dependent on recombination rate. They also reported that the deletion bias was not significantly correlated with recombination rate, observations that are at odds with our findings from the human genome. In Text S1 we show that primate ancestral repeats have a lower deletion rate and a lower deletion bias than more recently evolved repeats in the human lineage. We hypothesize that this is because of an ascertainment bias in the analysis of ARs since sequences that can be aligned over large evolutionary distances are less likely to harbor deletions. Moreover, since ancestral LINEs shared by human, chimpanzee, and macaque comprise less than 10% of total amount of LINEs in the human genome, they will have relatively limited influence on overall patterns inferred from analyses of present-day repeats.

Although transposable elements have emerged as a widely used sequence category for inferences of neutral rates and patterns of nucleotide substitution (e.g. [74]), as well as of insertion and deletion [75], a final cautionary note could be added. For example, it might be argued that the presence of undetected and active subfamilies originating from a single master sequence would violate the assumption of independent divergence of individual elements from the presumed master sequence. This could inflate estimates of divergence within individual LINE subfamilies. However, our results were not affected by restricting the analyses to indel events that were only seen once. This also excludes the possibility of concerted evolution from frequent gene conversion affecting our results. Moreover, unless the genomic distribution of repeats spreading from incorrectly inferred dead-on-arrival elements would be non-random with respect to recombination, the occurrence of undetected subfamilies is anyway unlikely to affect our conclusions. Finally, we note that the chronological order of activity of different LINE subfamilies as revealed by patterns of nested LINEs is entirely congruent with the relative age of subfamilies as revealed by divergence between individual elements and master sequences (Figure S4).

## Methods

### Sequence data

Sequence alignments of LINEs and their master sequences from zebra finch (taeGut1), chicken (galGal3), and human (hg18) were downloaded from the Repeatmasker homepage (http://www.repeatmasker.org/PreMaskedGenomes.html) [76]. These repeat elements had been identified using Repeatmasker 3.2.7 or 3.2.8 with the reference sequences and annotations of Repbase update 20090604 [77]. We excluded repeats located within exons and repeats of unassigned contigs (contigs with an unknown location in the genome). Data on sex-averaged recombination rates were obtained from [12] for human, from [13] for chicken, and from [14] for zebra finch.

Since SINEs constitute only a small proportion of all transposable elements in avian genomes [3], we limited the study to LINEs. We did not include DNA transposons since their cut-and-paste mechanism for transposition prohibits an unbiased analysis of insertion and deletion events within repeats. LTR retrotransposons were also excluded because solo-LTR elements, the product of intra-strand recombination, can bias divergence estimates.

### Data analysis

We concatenated all LINEs together with their aligned master sequences within 1 Mb windows. The insertion and deletion rates within transposable elements were calculated by dividing the length sum of insertions or deletions (in bp) by the length sum of transposable elements within the window in question. The deletion bias was calculated by dividing the number of deleted nucleotides by the number of inserted nucleotides within each window. Substitution rate of LINEs was calculated by using the baseml program in PAML4.4 [78]. To be able to take possible biased distribution of different LINE subfamilies (with different age profiles) across the genome into account, divergence was normalized by the relative age of each subfamily using the TinT program which counts the frequency of nested transposable elements [79]. Divergence of each window was normalized by the following equation:
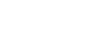
where 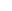 is the divergence, 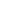 is the relative time of the maximal activity of subfamily *i* (note that a low t_i_ value indicates a high age), 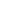 is number of LINEs in a window, and 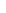 is the mean value of t_i_ for all subfamilies in the genome.

It might be argued that analyses of repeat elements from two avian genomes cannot be seen as independent samples if elements inserted before the split of chicken and zebra finch. We therefore made separate analyses involving recombination rate using galliform-specific (chicken) and passeriform-specific (zebra finch) subfamilies of repeats, respectively. The results from these lineage-specific analyses were very similar to the full data set and are not reported.

Since several parameters were not normally distributed we used Kendall's rank tests for correlation analyses. All statistical analyses were performed in the R platform (http://www.r-project.org). Mixed model analysis was performed in order to control for chromosome identity using the lme4 package [80]. We then used the pvals.fnc function that calculated *p*-values based on the *t* statistic, with the upper bound for the number of degrees of freedom.

### Comparison of autosomes and sex chromosomes

Non-parametric bootstrapping was performed in order to compare the sequence length of LINEs between sex chromosome and autosomes. The sequence length of each LINE was collected based on stratified random sampling and the difference in the mean LINE length between pairs of randomly grouped samples was used to test the null hypothesis. Bootstrapping was performed 10,000 times and significance level (*p* value) was obtained by calculating the proportion of replicates that had higher mean length difference between random categories than the real categories.

Comparison of the deletion bias between sex chromosome and autosomes was also tested using non-parametric bootstrapping and stratified random sampling. The difference in mean deletion bias between two categories of replicates was calculated by:
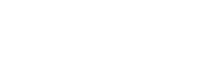
where D* and I* are the respective number of deleted and inserted sites from a randomly chosen LINE using stratified sampling, *a* is the number of LINEs in a single category, and *n* is the number of LINEs in both categories. Bootstrapping was performed 10,000 times and significance level (*p* value) was obtained by calculating the proportion of replicates that had higher (or lower) 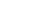 than the difference of the mean deletion bias from the real dataset.

### Modeling of the effect of the deletion bias on genome size

Change in sequence length can be expressed by the exponential decay function:
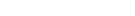
where *f(x)* is the length of neutrally evolving sequence, *r* is the rate parameter for an exponential decay function, and *t* is time. *r* was calculated from the change in sequence length over a given time period defined by the substitution rate using:
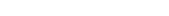
where *D*, *I* and *S* are deletion, insertion and substitution rates, respectively. This gives:
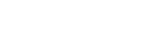

*D*, *I* and *S* of the chicken lineage after the split between chicken and turkey were calculated from 8,328 ancestral CR1 sequences identified in whole-genome alignment of chicken, turkey and zebra finch [29]. This identification was based on the Repeatmasker output file (http://www.repeatmasker.org/genomes/galGal3/galGal3.fa.out.gz) of the chicken genome, using in house perl programs. We excluded alignments where sequence length of turkey or zebra finch was shorter than 80% of the alignment length in order to minimize the effect from spurious sequence originating from non-repetitive CR1 flanking sequences. Ancestral CR1 elements were concatenated within each window, followed by the estimation of divergence in the chicken lineage. Genome-wide divergence was then estimated from the weighted divergences of each window according to the length of alignments. Confidence intervals were calculated from bootstrapping with 1,000 replicates.

## Supporting Information

Figure S1 Density-histogram of the size distribution of small insertions and deletions (bp) in (a, b) chicken, (c, d) zebra finch, and (e, f) human.
(TIF)

Figure S2 Simulated exponential decay curve showing the change in sequence length over time. The *x*-axis is the substitution rate, a proxy for time, and *y*-axis is the relative sequence length remaining after time *x*. 1,000 times of bootstrap re-sampling of ancestral repeats were performed to estimate the rate parameter. The solid curve is the mean rate parameter and the dashed curves represent the 95% confidence interval.
(TIF)

Figure S3 Overview of the net effect on sequence length of insertions (a–c) and deletions (d–f) of different size in the investigated species. Indel events are classified as small (1–2 bp), intermediate (3–20 bp) and long (>20 bp).
(TIF)

Figure S4 Correlation between divergence (sum of substitution, deletion, and insertion rates) estimated from alignment of individual repeat element and master sequences and the Tn value calculated from nested transposable elements using TinT program (Churakov et al. 2010). Each point represents a single LINE subfamily. The Kendall tau rank correlation coefficient (τ) for chicken, zebra finch, and human is −0.62, −0.67, and −0.74, respectively. The nested analysis builds on the principle that, for example, subfamily A should have been active prior to subfamily B if elements from subfamily B are found nested within elements from subfamily A, but not vice versa.
(TIF)

Table S1 Strength (correlation coefficient, τ) and statistical significance (*p*) of Kendall's rank correlations between recombination rate and various genomic parameters in non-overlapping 5 Mb windows.
(DOC)

Table S2 Statistics showing the fixed effect of log-transformed recombination rate on various genomic parameters after controlling for chromosomal identity. *t*-values were calculated by a mixed model implemented in the lme4 package in R. We used the pvals.fnc function that calculates *p*-values based on the *t* statistic with the upper bound for the number of degrees of freedom.
(DOC)

Table S3 Statistics for the correlation between recombination rate and deletion rate, and between recombination rate and deletion bias, for individual chromosomes in all three studied species. *p*-values are adjusted to take multiple testing into account according to Benjamini & Hochberg (1995)^a^.
(DOC)

Table S4 Comparison of the occurrence of human insertions and deletions with minor allele frequency categorized as rare (<0.05) or common (>0.05). Allele frequency data are from Mills et al. (2011).
(DOC)

Text S1 Deletion bias in human LINEs in relation to their age.
(DOC)
