# Supplementary figures and images for: Recombination Drives Vertebrate Genome Contraction
Source: PLoS Genet. 2012 May 3;8(5):e1002680. doi: 10.1371/journal.pgen.1002680 (PMC3342960; doi:10.1371/journal.pgen.1002680)

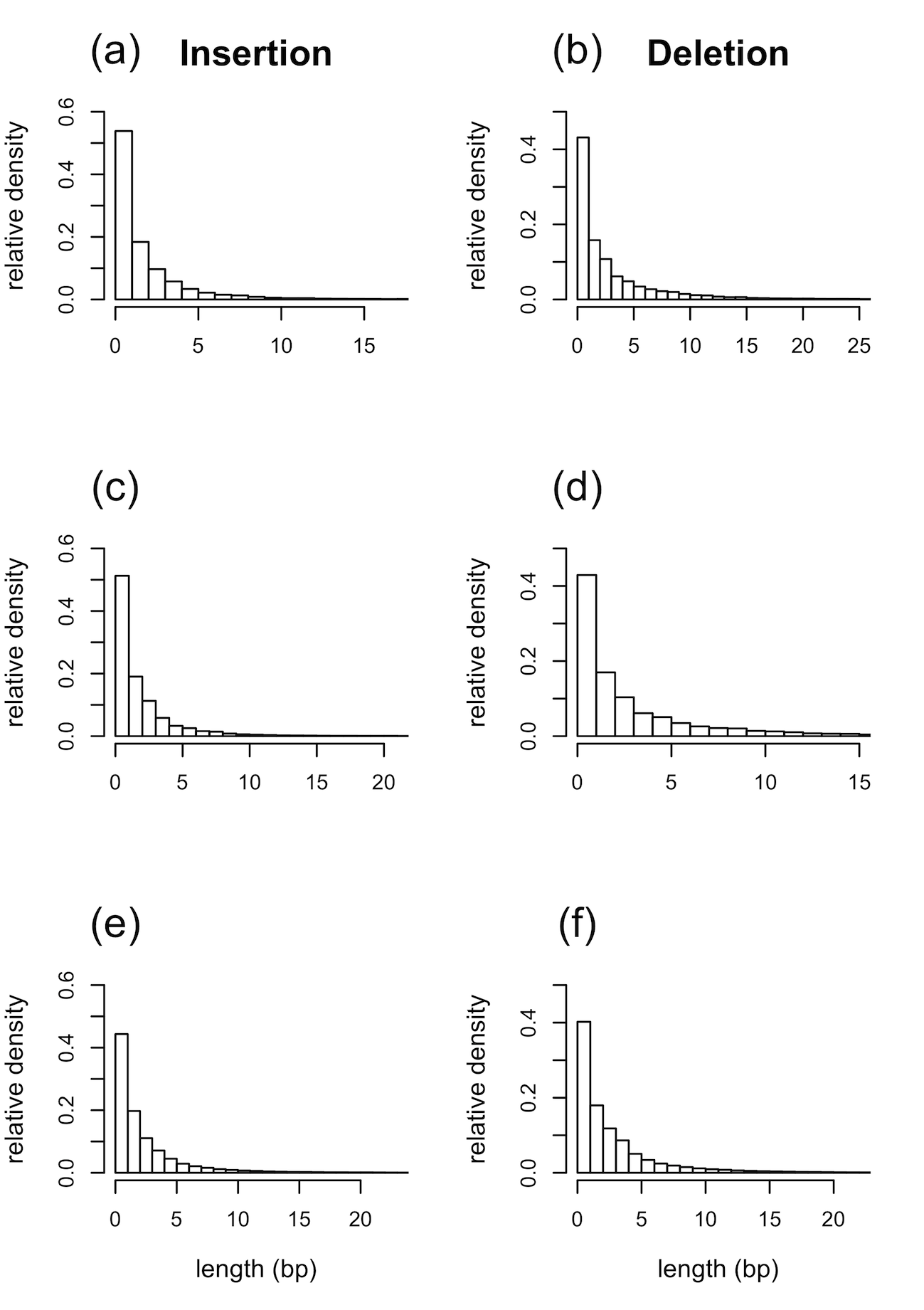

Supplement: Figure S1 — Density-histogram of the size distribution of small insertions and deletions (bp) in (a, b) chicken, (c, d) zebra finch, and (e, f) human. (TIF) [file pgen.1002680.s001.tif]

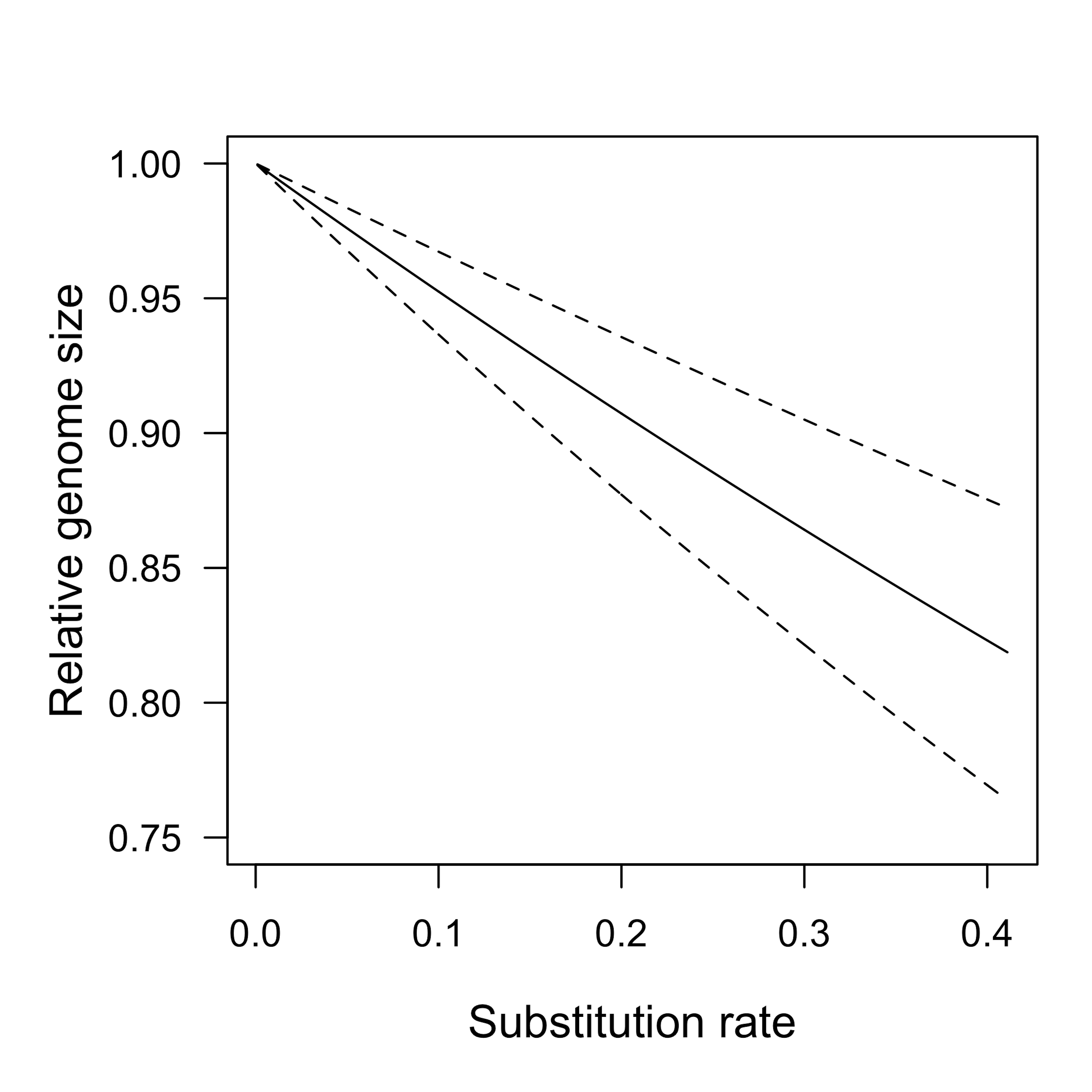

Supplement: Figure S2 — Simulated exponential decay curve showing the change in sequence length over time. The x-axis is the substitution rate, a proxy for time, and y-axis is the relative sequence length remaining after time x. 1,000 times of bootstrap re-sampling of ancestral repeats were performed to estimate the rate parameter. The solid curve is the mean rate parameter and the dashed curves represent the 95% confidence interval. (TIF) [file pgen.1002680.s002.tif]

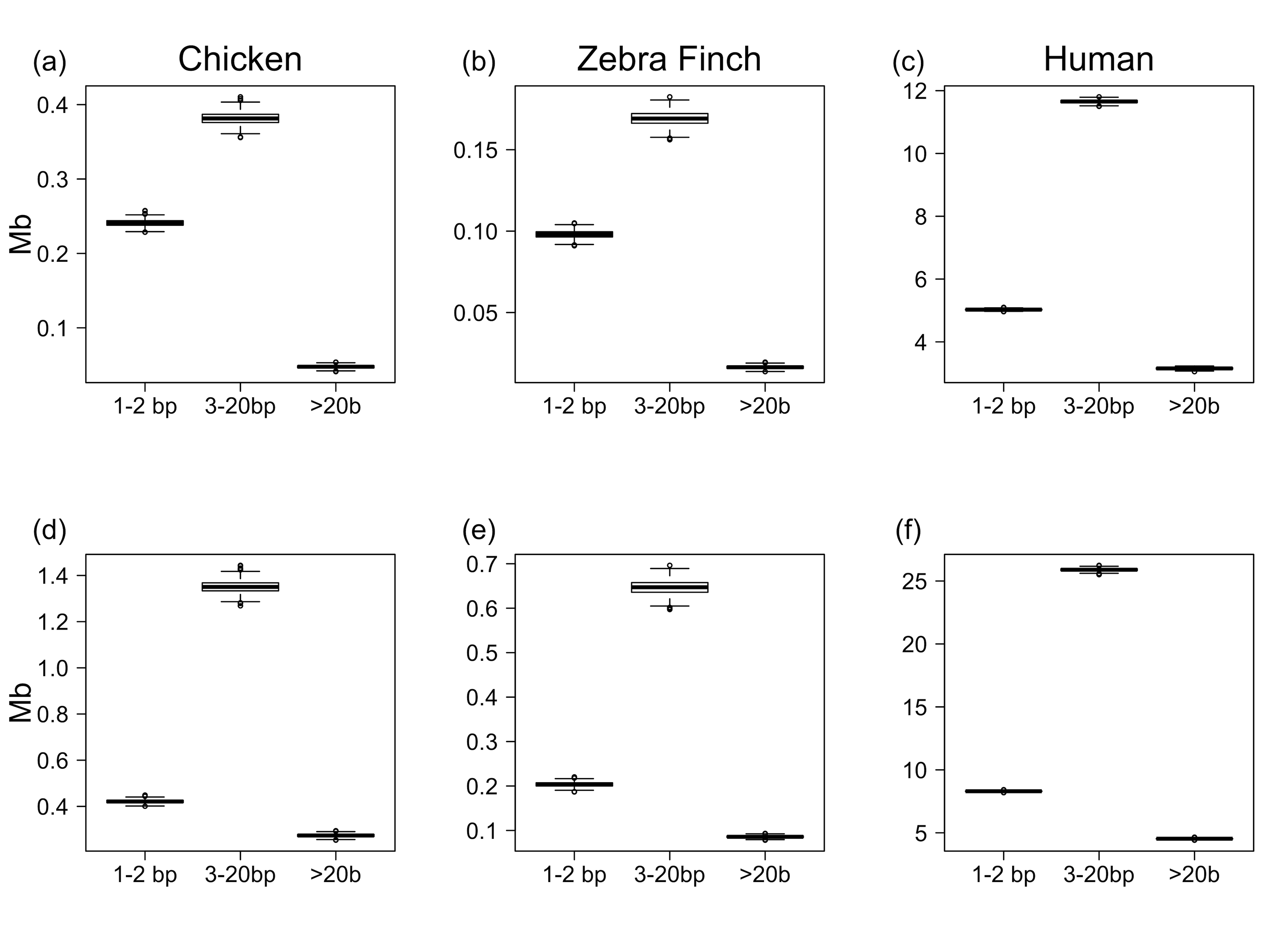

Supplement: Figure S3 — Overview of the net effect on sequence length of insertions (a–c) and deletions (d–f) of different size in the investigated species. Indel events are classified as small (1–2 bp), intermediate (3–20 bp) and long (>20 bp). (TIF) [file pgen.1002680.s003.tif]

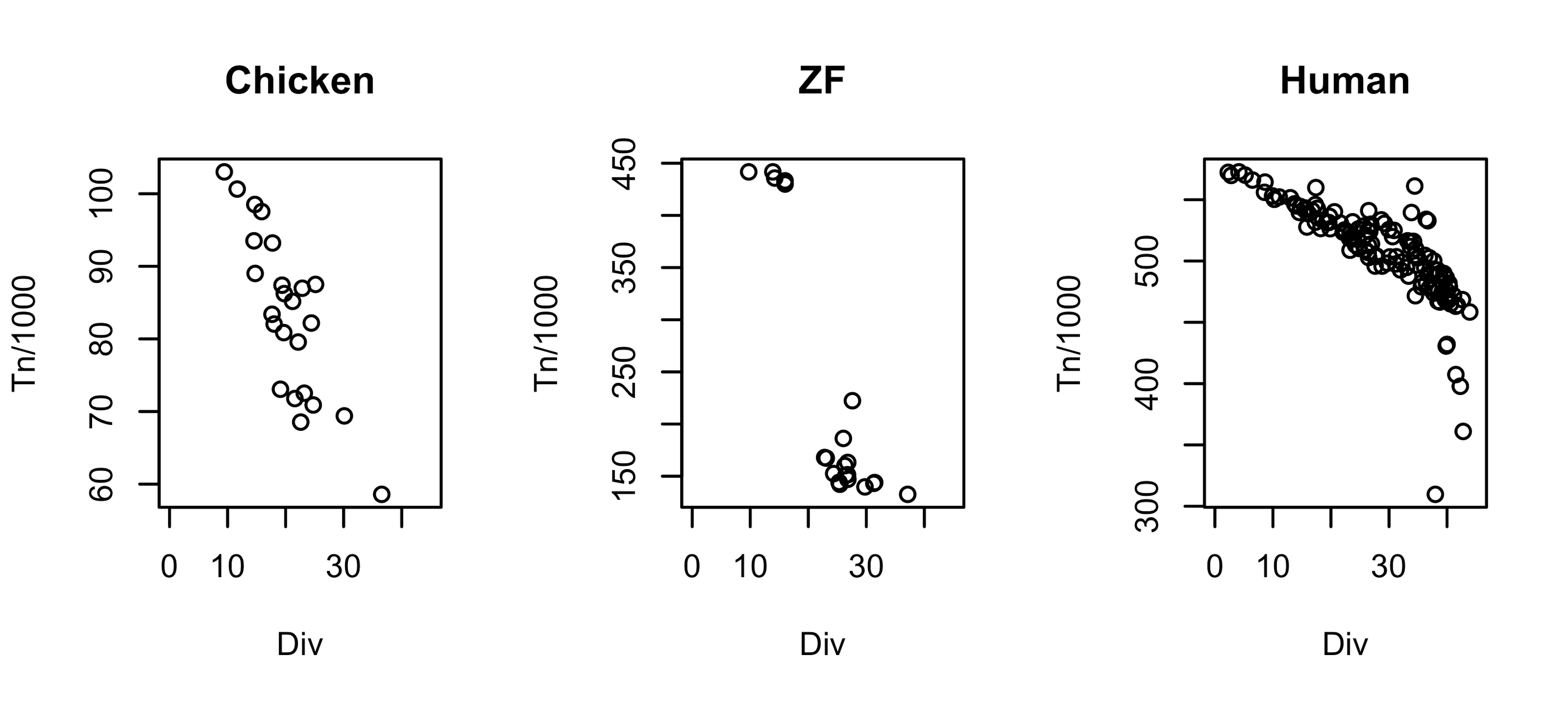

Supplement: Figure S4 — Correlation between divergence (sum of substitution, deletion, and insertion rates) estimated from alignment of individual repeat element and master sequences and the Tn value calculated from nested transposable elements using TinT program (Churakov et al. 2010). Each point represents a single LINE subfamily. The Kendall tau rank correlation coefficient (τ) for chicken, zebra finch, and human is −0.62, −0.67, and −0.74, respectively. The nested analysis builds on the principle that, for example, subfamily A should have been active prior to subfamily B if elements from subfamily B are found nested within elements from subfamily A, but not vice versa. (TIF) [file pgen.1002680.s004.tif]
