# Supplementary material for: Recombination Drives Vertebrate Genome Contraction
Source: PLoS Genet. 2012 May 3;8(5):e1002680. doi: 10.1371/journal.pgen.1002680 (PMC3342960; doi:10.1371/journal.pgen.1002680)
Supplement: Table S1 — Strength (correlation coefficient, τ) and statistical significance (p) of Kendall's rank correlations between recombination rate and various genomic parameters in non-overlapping 5 Mb windows. (DOC) [file pgen.1002680.s005.doc]

**Table S1**. Strength (correlation coefficient, τ) and statistical significance (*p*) of Kendall’s rank correlations between recombination rate and various genomic parameters in non-overlapping 5 Mb windows.

|  | Chicken | | Zebra finch | |
| --- | --- | --- | --- | --- |
|  | τ | *p* | τ | *p* |
| Intron length | -0.34 | < 0.001 | -0.29 | < 0.001 |
| First intron length | -0.27 | < 0.001 | -0.19 | 0.009 |
| Length of individual LINEs | -0.43 | < 0.001 | -0.53 | < 0.001 |
| Length of unique sequence within introns | -0.31 | < 0.001 | -0.29 | < 0.001 |
| Intergenic spacer length | -0.40 | < 0.001 | -0.23 | < 0.001 |
| Gene density | 0.37 | < 0.001 | 0.24 | < 0.001 |
| Deletion rate | 0.33 | < 0.001 | 0.45 | < 0.001 |
| Deletion bias | 0.27 | < 0.001 | 0.36 | < 0.001 |
| Net change in sequence length | 0.34 | < 0.001 | 0.47 | < 0.001 |
