# Supplementary material for: Recombination Drives Vertebrate Genome Contraction
Source: PLoS Genet. 2012 May 3;8(5):e1002680. doi: 10.1371/journal.pgen.1002680 (PMC3342960; doi:10.1371/journal.pgen.1002680)
Supplement: Table S2 — Statistics showing the fixed effect of log-transformed recombination rate on various genomic parameters after controlling for chromosomal identity. t-values were calculated by a mixed model implemented in the lme4 package in R. We used the pvals.fnc function that calculates p-values based on the t statistic with the upper bound for the number of degrees of freedom. (DOC) [file pgen.1002680.s006.doc]

**Table S2**. Statistics showing the fixed effect of log-transformed recombination rate on various genomic parameters after controlling for chromosomal identity. *t*-values were calculated by a mixed model implemented in the lme4 package in R. We used the pvals.fnc function that calculates *p*-values based on the *t* statistic with the upper bound for the number of degrees of freedom.

|  | Chicken | | Zebra finch | | Human | |
| --- | --- | --- | --- | --- | --- | --- |
|  | *t* | *p* | *t* | *p* | *t* | *P* |
| Intron length | -3.164 | 0.002 | -1.964 | 0.050 | -0.179 | 0.858 |
| First intron length | -3.827 | < 0.001 | -0.563 | 0.574 | 1.059 | 0.290 |
| Length of individual LINEs | -12.01 | < 0.001 | -8.54 | < 0.001 | -9.10 | < 0.001 |
| Length of unique sequence within introns | -2.822 | 0.005 | -1.969 | 0.049 | 0.612 | 0.541 |
| Intergenic spacer length | -12.61 | < 0.001 | -1.314 | 0.189 | -2.473 | 0.014 |
| Gene density | 9.472 | < 0.001 | 2.119 | 0.035 | -0.663 | 0.508 |
| Deletion rate | 4.67 | < 0.001 | 10.66 | < 0.001 | 11.66 | < 0.001 |
| Insertion rate | -1.41 | 0.159 | 0.11 | 0.910 | 8.11 | < 0.001 |
| Deletion bias | 5.16 | < 0.001 | 6.22 | < 0.001 | 5.56 | < 0.001 |
