# Supplementary material for: Recombination Drives Vertebrate Genome Contraction
Source: PLoS Genet. 2012 May 3;8(5):e1002680. doi: 10.1371/journal.pgen.1002680 (PMC3342960; doi:10.1371/journal.pgen.1002680)
Supplement: Table S3 — Statistics for the correlation between recombination rate and deletion rate, and between recombination rate and deletion bias, for individual chromosomes in all three studied species. p-values are adjusted to take multiple testing into account according to Benjamini & Hochberg (1995)a. (DOC) [file pgen.1002680.s007.doc]

**Table S3.** Statistics for the correlation between recombination rate and deletion rate, and between recombination rate and deletion bias, for individual chromosomes in all three studied species. *p*-values are adjusted to take multiple testing into account according to Benjamini & Hochberg (1995)^a^.

| **Species** | **Chr.** | **Deletion rate** | | **Deletion bias** | |
| --- | --- | --- | --- | --- | --- |
|  |  | τ | *p* | τ | *p* |
| **Chicken** | |  |  |  |  |
|  | 1 | -0.0003 | 0.9952 | 0.0996 | 0.2361 |
|  | 2 | 0.0958 | 0.2404 | 0.1037 | 0.2361 |
|  | 3 | 0.1116 | 0.2404 | 0.0826 | 0.3628 |
|  | 4 | 0.1765 | 0.1018 | 0.0246 | 0.8095 |
|  | 5 | 0.0741 | 0.5547 | 0.1340 | 0.2873 |
|  | 6 | -0.0334 | 0.9050 | 0.0620 | 0.8095 |
|  | 7 | 0.1508 | 0.4193 | -0.0121 | 0.9166 |
|  | 8 | 0.3045 | 0.1018 | 0.2860 | 0.2361 |
|  | 9 | 0.1377 | 0.5547 | 0.1449 | 0.5297 |
|  | 10 | 0.0421 | 0.9050 | 0.0737 | 0.8095 |
|  | 11 | 0.1619 | 0.5547 | 0.2667 | 0.2668 |
| **Zebra finch** | |  |  |  |  |
|  | 1 | 0.1732 | 0.0264 | 0.2403 | 0.0048 |
|  | 3 | 0.1848 | 0.0220 | 0.1912 | 0.0164 |
|  | 4 | 0.0715 | 0.4284 | 0.1622 | 0.1444 |
|  | 5 | 0.2313 | 0.0264 | 0.1323 | 0.2502 |
|  | 6 | 0.4300 | 0.0048 | 0.0811 | 0.6881 |
|  | 7 | 0.1558 | 0.2078 | 0.2162 | 0.1444 |
|  | 8 | 0.2246 | 0.1751 | -0.0725 | 0.7326 |
|  | 9 | 0.2533 | 0.1275 | 0.0400 | 0.7993 |
| **Human** | |  |  |  |  |
|  | 1 | 0.1791 | 0.0004 | 0.0635 | 0.4082 |
|  | 2 | 0.1772 | 0.0006 | 0.0131 | 0.9073 |
|  | 3 | 0.1693 | 0.0033 | 0.0749 | 0.4082 |
|  | 4 | 0.2970 | 0.0000 | 0.1050 | 0.1889 |
|  | 5 | 0.3484 | 0.0000 | 0.1911 | 0.0132 |
|  | 6 | 0.3363 | 0.0000 | 0.1332 | 0.0941 |
|  | 7 | 0.1555 | 0.0130 | 0.1594 | 0.0517 |
|  | 8 | 0.2628 | 0.0000 | 0.1237 | 0.1529 |
|  | 9 | 0.0124 | 0.8484 | -0.0017 | 0.9794 |
|  | 10 | 0.1390 | 0.0471 | 0.1527 | 0.0941 |
|  | 11 | 0.1730 | 0.0142 | 0.0721 | 0.4437 |
|  | 12 | 0.0938 | 0.2151 | 0.0723 | 0.4437 |
|  | 13 | 0.2858 | 0.0006 | 0.0766 | 0.5099 |
|  | 14 | 0.2211 | 0.0115 | 0.1594 | 0.1529 |
|  | 15 | -0.0503 | 0.6695 | 0.0674 | 0.5664 |
|  | 16 | 0.0451 | 0.6695 | -0.0982 | 0.4437 |
|  | 17 | -0.1096 | 0.2627 | 0.0311 | 0.8496 |
|  | 18 | 0.0492 | 0.6695 | -0.0143 | 0.9567 |
|  | 19 | 0.1181 | 0.3042 | 0.1055 | 0.4437 |
|  | 20 | -0.0271 | 0.8036 | -0.0447 | 0.8086 |
|  | 21 | 0.0575 | 0.7383 | -0.0713 | 0.8086 |
|  | 22 | 0.0859 | 0.6552 | 0.0036 | 0.9794 |

**^a^** Benjamini, Y., and Hochberg, Y. (1995). Controlling the false discovery rate: a practical and powerful approach to multiple testing. *Journal of the Royal Statistical Society Series* B, **57**, 289–300.
