# Supplementary material for: Recombination Drives Vertebrate Genome Contraction
Source: PLoS Genet. 2012 May 3;8(5):e1002680. doi: 10.1371/journal.pgen.1002680 (PMC3342960; doi:10.1371/journal.pgen.1002680)
Supplement: Table S4 — Comparison of the occurrence of human insertions and deletions with minor allele frequency categorized as rare (<0.05) or common (>0.05). Allele frequency data are from Mills et al. (2011). (DOC) [file pgen.1002680.s008.doc]

**Table S4**. Comparison of the occurrence of human insertions and deletions with minor allele frequency categorized as rare (<0.05) or common (>0.05). Allele frequency data are from Mills et al. (2011)

|  | Deletion | | | Insertion | | | Chi-square | *p* |
| --- | --- | --- | --- | --- | --- | --- | --- | --- |
|  | Common | Rare | % rare | Common | Rare | % rare |
| Intron | 1548 | 501 | 24.5 | 1501 | 371 | 19.8 | 12.14 | <0.001 |
| Intergenic | 2030 | 603 | 23.0 | 2089 | 556 | 21.0 | 2.72 | 0.099 |
| LINE | 76 | 13 | 14.6 | 60 | 18 | 23.1 | 1.97 | 0.160 |
