# Supplementary material for: Recombination Drives Vertebrate Genome Contraction
Source: PLoS Genet. 2012 May 3;8(5):e1002680. doi: 10.1371/journal.pgen.1002680 (PMC3342960; doi:10.1371/journal.pgen.1002680)
Supplement: Text S1 — Deletion bias in human LINEs in relation to their age. (DOC) [file pgen.1002680.s009.doc]

**Supplementary Text 1.** We identified 152,060 full-length ancestral LINEs from whole-genome alignments of human, chimpanzee, and macaque in the UCSC genome browser (http://genome.ucsc.edu/). This corresponds to 9.43% of total number of LINEs in human genome. Deletion and insertion rates of ancestral repeats were estimated by alignment to master sequences in concatenated 5 Mb windows, as described in the main text. We find that both insertion and deletion rates are positively correlated with recombination rate (τ = 0.15 and 0.12 for insertion and deletion, respectively; *p* < 0.001 for both), similar to what we found using all LINE sequences (see main text). However, the deletion bias in ancestral LINEs did not show a significant correlation with recombination rate (τ = -0.01; *p* = 0.745), which contrasts to what we found for all LINEs (τ = 0.12; *p* < 0.001). As divergence estimates in ancestral repeats are obviously higher than in repeats inserted in the human lineage subsequent to the split from the macaque and chimpanzee lineages, relative measures of deletion and insertion rates can be obtained by taking the ratio of the rate of deletion/insertion and substitution. For deletions, this ratio was significantly lower in ancestral LINEs than in all LINEs (0.1384 vs. 0.1658; Wilcoxon rank sum test, p<0.001), while for insertions the ratio was slightly but significantly higher in ancestral LINEs than in all LINES (0.0861 vs. 0.0839; *p* < 0.023). As a consequence, the deletion bias was lower in ancestral LINEs than in all LINEs (1.637 vs. 1.983; *p* < 0.001). It is possible that the lower deletion bias in ancestral LINEs can explain why Kvikstad and colleagues (2007) failed to find a correlation with recombination rate.

There are several alternative possibilities to why the deletion rate, and thereby the deletion bias, is lower in ancestral LINEs. For example, if some nucleotides are more prone to be deleted than others, and thus relatively quickly are removed, the “mean” deletion rate should be expected to be lower for older LINEs. Moreover, genomic regions with relatively high deletion rates are likely to be underrepresented in whole-genome alignments due to difficulties for alignment algorithm.
